# Comparative transcriptome analysis of two *Daphnia galeata* genotypes displaying contrasting phenotypic variation induced by fish kairomones in the same environment of the Han River, Korea

**DOI:** 10.1186/s12864-023-09701-x

**Published:** 2023-10-02

**Authors:** Tae-June Choi, Seung-Min Han, Adeel Malik, Chang-Bae Kim

**Affiliations:** 1 Department of Biotechnology, Sangmyung University, Seoul, 03016 Republic of Korea; 2 Institute of Intelligence Informatics Technology, Sangmyung University, Seoul, 03016 Republic of Korea

**Keywords:** Predator-induced response, Phenotypic plasticity, Transcriptome, RNA-seq, *Daphnia galeata*

## Abstract

**Background:**

Phenotypic plasticity is a crucial adaptive mechanism that enables organisms to modify their traits in response to changes in their environment. Predator-induced defenses are an example of phenotypic plasticity observed across a wide range of organisms, from single-celled organisms to vertebrates. In addition to morphology and behavior, these responses also affect life-history traits. The crustacean *Daphnia galeata* is a suitable model organism for studying predator-induced defenses, as it exhibits life-history traits changes under predation risk. To get a better overview of their phenotypic plasticity under predation stress, we conducted RNA sequencing on the transcriptomes of two Korean *Daphnia galeata* genotypes, KE1, and KB11, collected in the same environment.

**Results:**

When exposed to fish kairomones, the two genotypes exhibited phenotypic variations related to reproduction and growth, with opposite patterns in growth-related phenotypic variation. From both genotypes, a total of 135,611 unigenes were analyzed, of which 194 differentially expressed transcripts (DETs) were shared among the two genotypes under predation stress, which showed consistent, or inconsistent expression patterns in both genotypes. Prominent DETs were related to digestion and reproduction and consistently up-regulated in both genotypes, thus associated with changes in life-history traits. Among the inconsistent DETs, transcripts encode vinculin (*VINC*) and protein obstructor-E (*OBST-E*), which are associated with growth; these may explain the differences in life-history traits between the two genotypes. In addition, genotype-specific DETs could explain the variation in growth-related life-history traits between genotypes, and could be associated with the increased body length of genotype KE1.

**Conclusions:**

The current study allows for a better understanding of the adaptation mechanisms related to reproduction and growth of two Korean *D. galeata* genotypes induced by predation stress. However, further research is necessary to better understand the specific mechanisms by which the uncovered DETs are related with the observed phenotypic variation in each genotype. In the future, we aim to unravel the precise adaptive mechanisms underlying predator-induced responses.

**Supplementary Information:**

The online version contains supplementary material available at 10.1186/s12864-023-09701-x.

## Background

Organisms are affected by a wide range of biotic and abiotic environmental factors, interacting, and adapting to environmental changes by phenotypic plasticity, evidenced through behavioral or physiological changes [1, 2]. Phenotypic plasticity refers to the capacity of genotypes to generate a range of phenotypes in response to environmental conditions, facilitating the survival and reproduction of organisms in diverse habitats [1, 2]. Phenotypic variation within a population is critical to its persistence, as less variation increases the risk of extinction [3, 4]. Genetic variation due to environmental change, gene flow, positive selection, mutation, and recombination can lead to loss or gain of phenotypic plasticity [5–8]. In addition, as phenotypic plasticity allows to locally adapt to a changed environment, if a population produces a phenotype more adapted to its environment, then the genotype of that population will contribute more to the genetic makeup of the entire population [9].

Among all environmental factors, predation is an important biological factor that maintains species diversity, organizes entire communities, and drives natural selection in populations [10, 11]. Vertebrate and invertebrate aquatic predators emit kairomones into the surrounding water, which can be detected by prey, eliciting predator-specific responses to reduce vulnerability [12, 13]. Adaptive strategies in response to predators, a typical example of phenotypic plasticity, include life-history changes, behavioral plasticity, and changes in morphological and physiological traits [14], and have been extensively studied across diverse *Daphnia* species [15, 16].

*Daphnia* species are small branchiopod crustaceans widely used as model organisms in ecology, evolution, and ecotoxicology [17, 18]. This genus primarily feeds on algae and is highly vulnerable to predation risk because it is the preferred food of many fish [17, 19]. In the genus *Daphnia*, broad alterations in morphology, behavior, and life-history traits have been observed in response to predation risk. *Daphnia* species have developed suitable survival and reproductive strategies in various aquatic environments and vegetation conditions, allowing them to inhabit regionally diverse underwater environments. The ability of *Daphnia* to locally adapt to different stressors has been demonstrated for fish as a vertebrate predator and *Chaoborus* as an invertebrate predator, for instance [20–22]. Such adaptations are associated with the maintenance of genetic diversity in each region and are related to intraspecific phenotypic variation. The distinct phenotypic responses observed among *Daphnia* populations may be due to unique colonization patterns, monopolization effects, and limited gene flow between populations, as well as significant genetic diversity [23]. In addition, one population of a *Daphnia* species can have individuals showing different genotypes; these genetic variations act as adaptive strategies to improve their ability to cope with environmental changes [9, 24]. Different genotypes have their own characteristics and survival strategies, allowing *Daphnia* populations to spread and adapt within their ecological range. Thus, studying genetic diversity within populations is essential for understanding the biodiversity and adaptive strategies of local ecosystems, as it plays an important role in developing different adaptive strategies and adapting to specific environmental conditions.

Despite previous reports on clonal variation in *Daphnia* [25, 26], previous studies have primarily drawn conclusions based on a single genotype (clonal line) rather than in ≥ 1 genotypes per population. Although intra-population variation is typically associated with population maintenance in response to predation pressure, the significance of such variation has rarely been studied. Previous studies have shown that when 16 *D. magna* genotypes from four different habitats were exposed to fish kairomones, response traits could vary between genotypes [20]. Moreover, Tams et al. found differences in life-history traits across 24 *D. galeata* genotypes within the same population and between populations when exposed to fish kairomones [9].

*D. galeata* is a relatively large-sized zooplankton found throughout the northern hemisphere and identified as a dominant plankton species in the Han River, Korea [27, 28]. It is also a member of the *Daphnia longispina* complex, which includes *D. longispina*, *D. cucullata*, *D. hyalina*, and *D. galeata*, and a widely distributed species, whose genetic diversity has been investigated in several countries in Asia, Europe, and North America [28–30]. Although this species does not exhibit substantial morphological changes or diel vertical migration in response to vertebrate predator cues, it exhibits marked phenotypic variation in life-history traits when facing predation risk [31]. Accordingly, *D. galeata* shows an adaptive strategy to maintain generations by producing more offspring when exposed to fish kairomones [32]. In addition, *Daphnia* can increase its body size in the presence of predators as a response to predator-induced stress [33]. A larger body size can provide advantages such as increased fecundity and improved competitive ability, which can help *Daphnia* thrive in predator-rich environments [33]. Conversely, *Daphnia* can also decrease its body size in response to predators [34, 35]. This adaptive response allows them to better avoid being detected and eaten by the predator. A smaller body size reduces visibility, making it more difficult for the predator to see and capture the prey. Previous studies have also shown that when *Daphnia* is exposed to fish kairomones, energy resources are preferentially allocated to somatic growth, increasing the rate at which neonates reach adulthood, thereby advancing their reproductive age [36]. These results show that the somatic growth rate is also related to reproduction [32, 37].

Extensive research has been conducted on phenotypic plasticity responses to predation stress in *Daphnia*, with efforts made to establish connections between these responses and genome-wide expression patterns. For example, Tams et al. performed transcriptional profiling of *D. galeata* exposed to fish kairomones and identified differentially expressed genes related to digestion and growth [32]. In addition, under vertebrate predation risk, *D. ambigua* also showed expression changes in genes involved in digestion, reproduction, and exoskeleton structure [38]. Another study identified up-regulated genes such as those encoding for the cuticle, vitellogenin, and others in response to invertebrate predation risk in *D. pulex* [22, 36]. However, the genetic basis of the appropriate functional responses associated with phenotypic plasticity in *Daphnia* species to predation risk is not well understood. Furthermore, it is not clear how different genotypes inhabiting the same environment exhibit different responses in their life-history traits under predation stress from a genotype-specific perspective.

The present study aimed to compare the phenotypic variation and associated transcriptional profiles of two *D. galeata* genotypes exposed to fish kairomones, which show different life-history traits despite inhabiting the same environment in the Han River, Korea. We applied a transcriptomic approach (RNA-seq) to compare the gene expression differences related to phenotypic variations between the two genotypes, followed by differential expression (DE), Gene Ontology (GO), and Kyoto Gene and Genome Encyclopedia (KEGG) pathway analyses. Additionally, network analysis was performed to infer the functions of the detected differentially expressed transcripts (DETs). Network-based research offers a structured approach to comprehensively analyze complex datasets and facilitate a holistic understanding of multiple interacting groups. The present results offer the foundation for a better understanding of the adaptation mechanisms related to the reproduction and growth of two Korean *D. galeata* genotypes under predation stress.

## Results

### Life-history traits

**Fig. 1:**
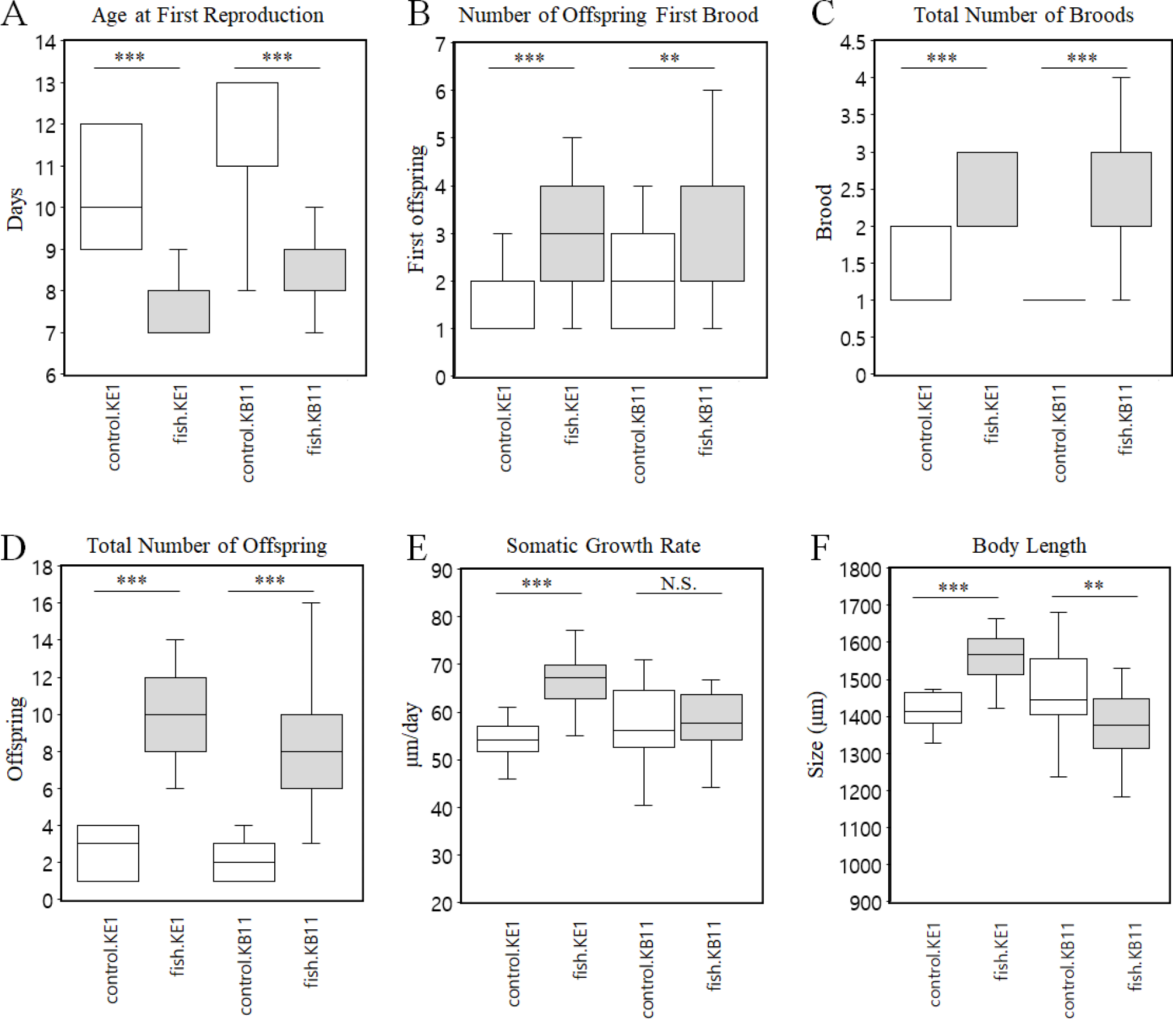
Box plots of reaction norms for selected life-history traits of two *D. galeata* genotypes (median +/− SD). A, Age at First Reproduction. B, Number of Offspring First Brood. C, Total Number of Broods. D, Total Number of Offspring. E, Somatic Growth Rate. F, Body Length. Control.KE1 indicates the control group (without fish kairomones) of genotype KE1 and fish.KE1 indicates the experimental group (fish kairomone-exposed) of genotype KE1. Control.KB11 indicates the control group (without fish kairomones) of genotype KB11 and fish.KB11 indicates the experimental group (fish kairomone-exposed) of genotype KB11. Stars indicate significant differences (*P.*adjust *** <0.001, ** <0.01, * <0.05, N.S., no significance) between fish kairomone-exposed and non-exposed in both genotypes

To compare the changes in life-history traits under predator stress in two *D. galeata* genotypes, six life-history traits related to reproduction and growth were measured. Reaction norms of life-history traits show intraspecific variation of life-history traits between the two *D. galeata* genotypes (Fig. 1). A one-way ANOVA showed that “fish kairomones” had a significant effect (*P* < 0.01) on the six life-history traits in both genotypes (Fig. 1). Both genotypes exhibited significant changes (*P* < 0.001) in life-history traits associated with reproduction, specifically decreasing the “Age at First Reproduction” in the presence of fish kairomones (Fig. 1A). In contrast, the “Number of Offspring First Brood,” “Total Number of Broods,” and “Total Number of Offspring” significantly increased (*P* < 0.05) in both genotypes in the presence of fish kairomones (Fig. 1B–D). Both genotypes exhibited similar patterns in life-history traits related to reproduction under fish kairomone exposure, which may be an adaptive strategy to produce more offspring and maintain generations by advancing reproductive age. However, the two genotypes exhibited contrasting patterns in terms of life-history traits associated with growth. In the life-history traits related to growth, “Somatic Growth Rate” of the genotype KE1 significantly increased (*P* < 0.001) in the presence of fish kairomones, and as a result, “Body Length” significantly increased (*P* < 0.01; Fig. 1E and F). Conversely, the “Somatic Growth Rate” of the genotype KB11 did not differ between fish kairomone-exposed and non-exposed conditions (Fig. 1E). However, “Body Length” was significantly decreased (*P* < 0.01) in the genotype KB11 (Fig. 1F). In summary, genotype KE1 exhibited an adaptive strategy by increasing the “Somatic Growth Rate” and “Body Length”, thereby promoting faster growth and then investing in high reproductive effort. In contrast, genotype KB11 decreased the “Body Length” to better avoid being detected and eaten by the predator. Hence, it can be observed that each genotype has a distinct adaptation strategy to cope with predation by fish.

### Datasets and *de novo* transcriptome assembly

RNA-seq was performed on the control and experimental groups of the two *D. galeata* genotypes. A total of 704,814,567 raw reads were generated from RNA-seq, of which 97.86% (689,735,869 reads) were retained after removing low-quality reads and trimming. Table S1 provides a summary of the RNA-seq data. A *de novo* transcriptome assembly generated a total of 186,084 transcripts, after removing redundant sequences, the longest transcripts from the CD-Hit clustering results were selected as unigenes, resulting in a total of 135,611 unigenes. Among them, the longest had a length of 28,597 bp while the shortest was 181 bp long. The mean GC content of unigenes was 40.7% (Table S2). The BUSCO estimation of completeness of the assembled transcripts showed that most orthologs among arthropods were represented in the assembled transcriptomes. In each dataset, 99.1% of complete orthologs belonging to the phylum Arthropoda were detected out of the 1013 BUSCO groups examined (Table S3). This includes both complete single copy and duplicates (putative paralogs or complete genes with multiple copies).

### Identification of coding regions and unigene functional annotation

A total of 135,611 unigenes were employed as query sequences for functional annotation, with TransDecoder identifying 89,738 unigenes having the longest open reading frames (ORFs) within the unigene datasets. Only ORFs that were at least 100 amino acids long were considered; sequences shorter than 100 residues were excluded. To identify ORFs with homology to known proteins, these transcripts were scanned against the UniProt and Pfam databases. The annotation result and corresponding accession numbers for all unigenes are provided in Table S4. Among 89,738 unigenes, a BLASTp-based homology search for *D. galeata* resulted in the annotation of 56,306 (62.74%) unigenes (Table S5). Similarly, about 75% and 66% of unigenes were mapped to the Pfam and eggNOG databases, respectively. In contrast, only 1,405 unigenes had KEGG annotations (Table S5). Overall, a total of 74,082 unigenes were annotated using various databases.

### DE and GO enrichment analysis

**Fig. 2:**
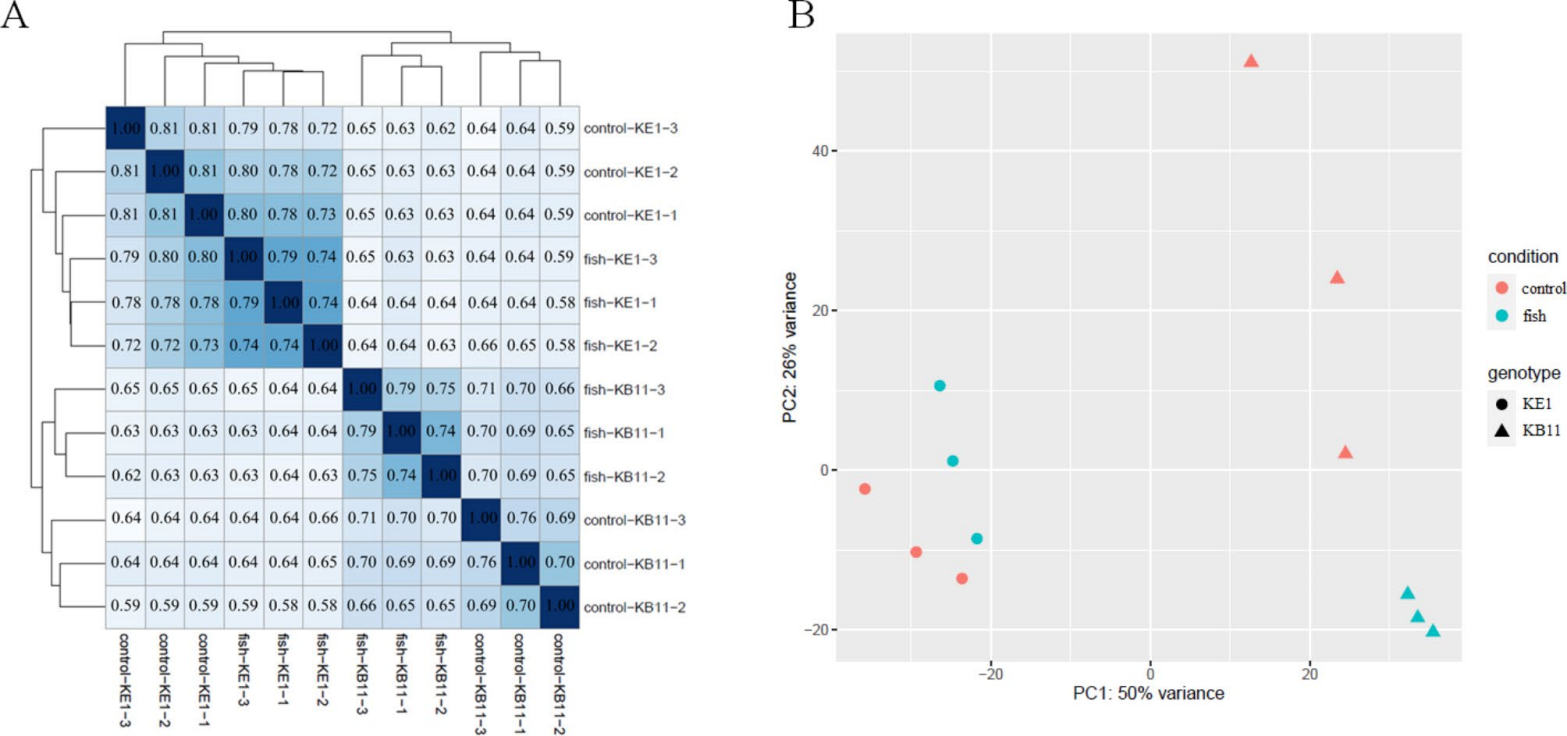
A, Pairwise correlation of all biological replicated RNA-seq samples in *D. galeata*. B, PCA plot of gene expression in *D. galeata* RNA-seq samples. Red, control group (without fish kairomones); Cyan, experimental group (fish kairomone-exposed). Circles, genotype KE1; Triangles, genotype KB11

Pearson correlation coefficient and principal component analysis (PCA) were performed to confirm the deviation of triplicated samples by visualizing differences in transcript expression (Fig. 2). The pearson correlation coefficient showed a strong correlation between samples for each genotype (Fig. 2A), and the PCA clearly clustered samples according to genotypes. The first principal component (PC 1) explained 50% of the variance between genotypes, and PC 2 explained 26% of the variance, an effect possible related to variance between replicates (Fig. 2B). Individually, each genotype revealed obvious clustering between control and experimental group (Figure S1). Therefore, we confirmed the consistency between triplicate samples, so the triplicate data was used for DE analysis.

**Fig. 3:**
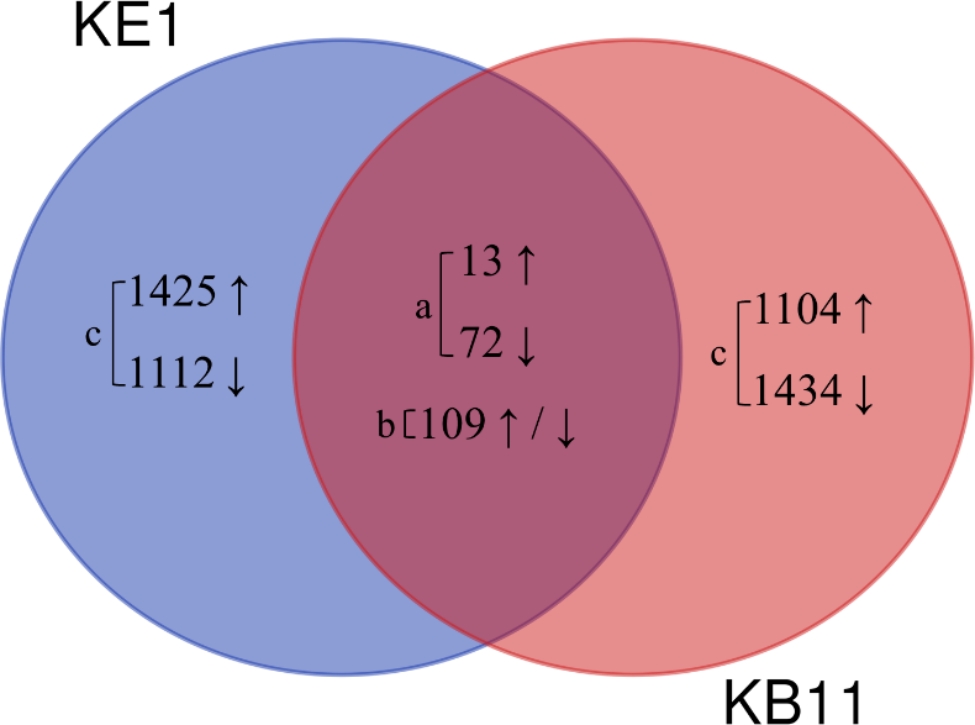
Venn diagram showing the number of DETs identified from each *D. galeata* genotype. **a**) Shared DETs showing consistent expression profiles across genotypes; **b**) Shared DETs showing inconsistent expression profiles across genotypes and; **c**) DETs uniquely identified in each genotype. ↑ and ↓ indicate up- and down-regulation, respectively

To understand the association between phenotypic variation and genotype-specific responses to fish kairomones, we selected and described DETs based on three criteria: (a) shared DETs that showed consistent expression profiles across both genotypes; (b) shared DETs that showed inconsistent expression profiles across both genotypes and; (c) DETs uniquely identified in each genotype (Table 1; Fig. 3). The complete list of DETs from both genotypes with detailed annotations is provided in Table S6. First, 194 DETs were shared among the two genotypes, 85 exhibited consistent expression profiles (e.g., up or downregulation) in both genotypes (Fig. 3 and Table S7). Consistently up-regulated DETs were homeobox protein cut (*CT*), dipeptidase 1 (*DPEP1*), vitellogenin (*VG*), and others (Table 1 and Table S7). In contrast, consistently down-regulated DETs were vitellogenin (*VG*), perlucin, and others (Table 1 and Table S7). Second, the remaining 109 DETs showed inconsistent expression (i.e., these transcripts exhibited contradictory expression patterns in both genotypes; Fig. 3 and Table S7). For example, vinculin (*VINC*) and protein obstructor-E (*OBST-E*) were up-regulated in KE1 but down-regulated in KB11 (Table 1 and Table S7). Similarly, the protein flightless-1 (*FLIL*) and dynamin (*SHI*) were down-regulated in KE1 but up-regulated in KB11 (Table 1 and Table S7). Finally, to associate the uniquely found DETs in each genotype with observed phenotypic variation, we compiled a list of 896 and 801 unique DETs in KE1 and KB11, respectively (Figure S2). To accomplish this, we initially excluded the DETs shared by both genotypes. Subsequently, we excluded DETs from each genotype that were annotated with the same Uniprot ID but displayed inconsistent expression patterns. In KE1, up-regulated DETs related to growth, including matrix metalloproteinase-2 (*MMP2*), dopamine D2-like receptor (*DOP2R*), and others were found; this was not the case in KB11 (Table 1 and Table S6). Conversely, down-regulated DETs related to growth, such as paxillin (*PXN*), muscle Lim protein Mlp84B (*MLP84B*), and others were found in KB11 but not in KE1 (Table 1 and Table S6).

**Table 1:** The list of consistent, inconsistent, and unique DETs associated with fish kairomones in each genotype

Criteira	Genotype	UniProtID	Effect	Descriptions
a) Consistent DETs	KE1/KB11	P10180	Up	Homeobox protein cut
		P31429	Up	Dipeptidase 1
		Q868N5	Up	Vitellogenin
		Q9N4J2	Down	Vitellogenin-3
		P82596	Down	Perlucin
b) Inconsistent DETs	KE1/KB11	O46037	Up/Down	Vinculin
		Q9VMM6	Up/Down	Protein obstructor-E
		Q24020	Down/Up	Protein flightless-1
		P27619	Down/Up	Dynamin
c) Unique DETs	KE1	Q8MPP3	Up	Matrix metalloproteinase-2
		Q8IS44	Up	Dopamine D2-like receptor
	KB11	P49024	Down	Paxillin
		Q24400	Down	Muscle LIM protein Mlp84B

**Fig. 4:**
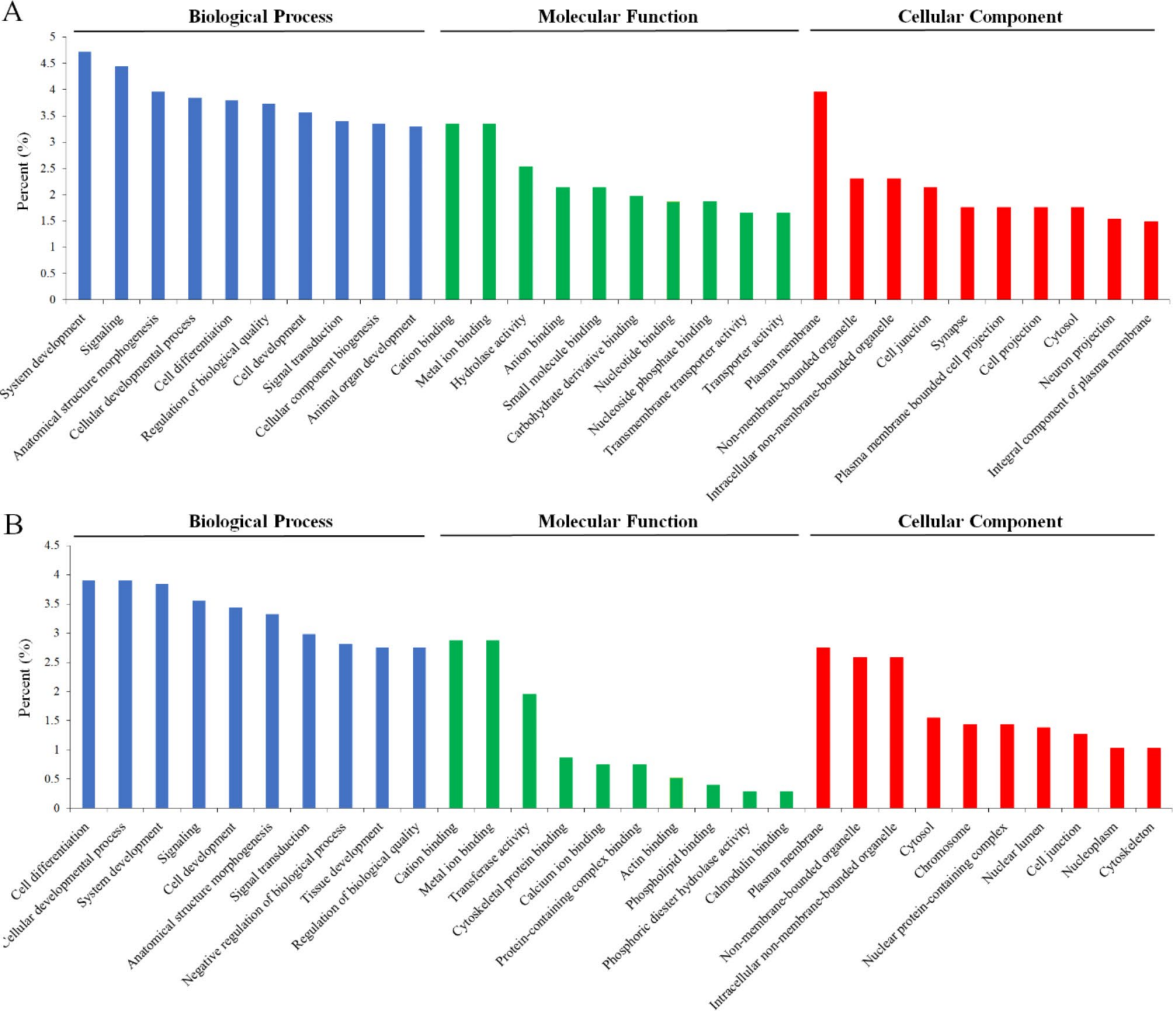
Gene ontology (GO) enrichment of DETs. The provided results display the top 10 processes within each GO category, encompassing biological processes (BP), molecular functions (MF), and cellular components (CC). A, genotype KE1. B, genotype KB11. Percentage (%) indicates the ratio of assigned genes to each GO term

Next, the enriched GO categories related to the biological process (BP), molecular function (MF), and cellular component (CC) were identified for the DETs. In KE1, GO terms related to development, such as “system development,” “cellular developmental process,” “cell development,” and “animal organ development” were among the top 10 enriched biological processes (Fig. 4A). In addition, 32 enriched GO BP were related to signaling and included regulation of signaling, cell-cell signaling, anterograde trans-synaptic signaling, trans-synaptic signaling, G protein-coupled receptor signaling pathway, and tachykinin receptor signaling pathway (Table S8). Most enriched GO MF were associated with binding of various molecular compounds (e.g., cation, metal ion, anion, small molecule, carbohydrate, etc.) or hydrolase and transmembrane transporter activities in KE1 (Fig. 4A). The GO CC showing the highest enrichment were mainly related to membranes (e.g., plasma membrane, integral component of plasma, etc.), cell junction, synapse, cell projection, cytosol, and neuron projection in KE1 (Fig. 4A).

Similarly, GO terms related to development were the enriched BP in the KB11 (Fig. 4B). In addition, 27 enriched GO BP related to signaling such as signal transduction, regulation of signaling, cell surface receptor signaling pathway, cell-cell signaling, anterograde trans-synaptic signaling, trans-synaptic signaling, hippo signaling, notch signaling pathway, and regulation of G protein-coupled receptor signaling pathway were detected in KB11 (Table S8). Interestingly, the tachykinin receptor signaling pathway was enriched only in KE1, while hippo signaling and notch signaling pathways were enriched only in KB11 (Table S8). In addition, the GO BP related to growth such as somatic muscle development, developmental growth, and cuticle development were enriched only in KE1, where “Somatic Growth Rate” and “Body Length” were significantly increased under fish kairomone exposure (Table S8). The GO MF of KB11 were found to be similar to those of KE1, with the most enriched categories associated with the binding of various molecular compounds such as cations, metal ions, anions, small molecules, carbohydrates, and more (Fig. 4B). We identified three down-regulated DETs, *MLP84B*, myosin heavy chain, muscle (*MHC*), and pupal cuticle protein Edg-78E (*EDG78E*), only in genotype KB11. These three DETs have been involved in MFs related to structural constituents of muscle or/and structural constituent of pupal chitin-based cuticle, and likely associated with the decrease in “Body Length” observed under fish kairomone exposure. Most enriched GO CC were related to membranes, cytosol, chromosome, nuclear protein-containing complex, nuclear lumen, cell junction, nucleoplasm, and cytoskeleton in KB11 (Fig. 4B).

### Enrichment analyses of KEGG pathways of DETs

The DETs were used for searching pathways that contribute to the alterations in life-history traits. Enrichment analysis of the pathway was conducted using the KEGG database. Table 2 provides an overview of the top 10 enriched KEGG pathways in KE1 and KB11, respectively; a complete list of pathways is provided in Table S9. Enriched categories with a corrected *p*-value < 0.05 were selected. The KEGG enrichment analysis identified 24 significant pathways of the DETs for KE1. Most were related to metabolic pathways, amino sugar and nucleotide sugar metabolism, AGE-RAGE signaling pathway in diabetic complications, and fatty acid metabolism. In addition, various metabolic (e.g., arachidonic acid, fructose and mannose, taurine, hypotaurine, etc.) and biosynthetic (e.g., fatty acid, steroid, and folate) pathways were enriched in KE1. Similarly, KEGG enrichment analysis identified 30 significant pathways for KB11, most related to autophagy - animal, protein processing in endoplasmic reticulum, metabolic pathways, AGE-RAGE signaling pathway, and amino sugar and nucleotide sugar metabolism in diabetic complications. Interestingly, four pathways, Wnt signaling pathway, FoxO signaling pathway, ECM-recepter interaction, and MAPK signaling pathway - fly were identified only in KB11.

**Table 2:** Top 10 enriched KEGG pathways for DETs of two *D. galeata* genotypes identified by RNA-seq. Only enriched categories with a *p*-value < 0.05 were selected

Genotype	Pathway Name	Pathway ID	Corrected *p*-value
KE1	Metabolic pathways	dme01100	1.94E-15
	Amino sugar and nucleotide sugar metabolism	dme00520	3.52E-12
	AGE-RAGE signaling pathway in diabetic complications	dme04933	2.70E-11
	Lysosome	dme04142	2.52E-09
	Fatty acid metabolism	dme01212	1.11E-06
	Neuroactive ligand-receptor interaction	dme04080	2.05E-06
	Fatty acid degradation	dme00071	6.91E-06
	Autophagy - animal	dme04140	1.88E-05
	Fatty acid biosynthesis	dme00061	1.97E-05
	Arachidonic acid metabolism	dme00590	2.25E-05
KB11	Autophagy - animal	dme04140	3.15E-20
	Protein processing in endoplasmic reticulum	dme04141	3.30E-13
	Metabolic pathways	dme01100	6.97E-06
	Wnt signaling pathway	dme04310	2.85E-05
	Phosphatidylinositol signaling system	dme04070	2.85E-05
	Ubiquitin mediated proteolysis	dme04120	7.03E-05
	AGE-RAGE signaling pathway in diabetic complications	dme04933	7.47E-05
	Amino sugar and nucleotide sugar metabolism	dme00520	7.47E-05
	FoxO signaling pathway	dme04068	7.70E-05
	Toll and Imd signaling pathway	dme04624	0.00089

### Interaction network of DETs

**Fig. 5:**
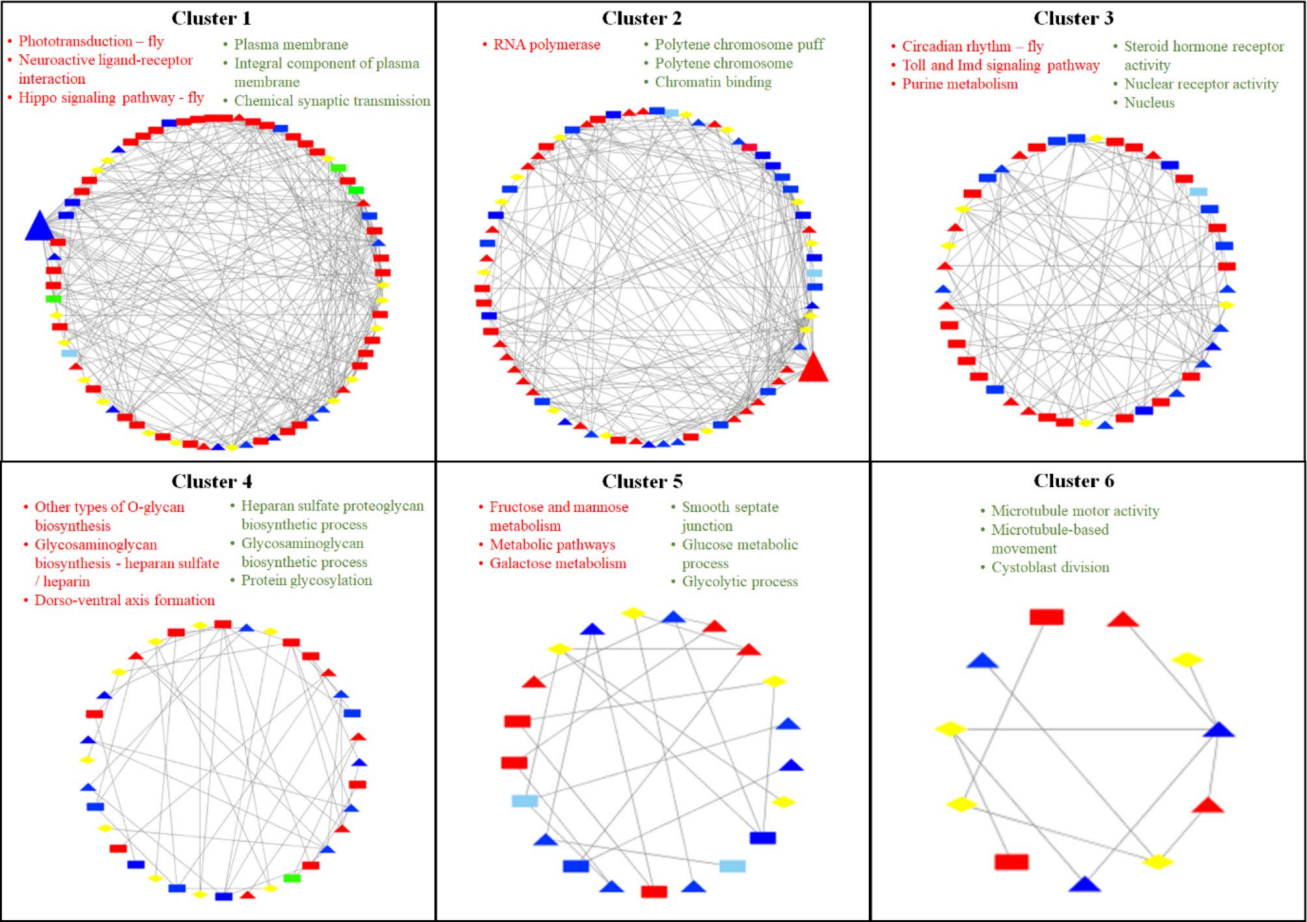
Interaction network of DETs detected in both genotypes. Red and blue nodes represent up- and down-regulated transcripts, respectively. Nodes in the rectangle and triangle indicate the genotype KE1 and KB11, respectively. Green and cyan squares represent up- and down-regulated transcripts in both genotypes. Yellow diamonds represent transcripts showing conflicting expression patterns in both genotypes. Large nodes in clusters 1 and 2 represent the hubs. The top 3 enriched KEGG pathways (red text) and GO terms (green text) are indicated for each cluster

There has been an increasing interest in utilizing biological networks to explore and understand significant biological phenomena [39]. Network-based investigations provide a comprehensive framework for gaining a holistic understanding of data, encompassing multiple interacting groups. The potential of community-based network analysis enables researchers to explore and compare a diverse range of proteins and their interactions within the identified modules or communities of the network [40]. Therefore, we attempted to identify the modular structure of the interaction network between DETs, thus dividing the network into functional six communities (clusters; Fig. 5).

To predict interactions between DETs, a non-redundant list of 2,119 DETs was compiled from both genotypes and mapped to STRING-DB using the common fruit fly (*Drosophila melanogaster*) as reference organism. Using the UniProt database to annotate unigenes, *Drosophila melanogaster*, exhibited the highest number of annotated unigenes among arthropods, leading to a subsequent network analysis using this species. Of these 2,119 DETs, only 338 were mapped to the fruit fly proteome. The resultant interaction network was subjected to community (cluster) detection to identify the functional modules. After removing clusters with < 10 nodes, the interaction network was divided into six main clusters comprising 257 nodes (DETs), and 1,314 edges (interactions between nodes; Fig. 5 and Table S10). The number of nodes in each cluster ranged between 11 (cluster 6) and 73 (cluster 1). The node degree of each node in the network ranged between 1 and 29; nodes with a maximum node degree ≥ 20 were considered as hubs. These hub nodes were identified to encode a tyrosine-protein kinase Src42A (*SRC42A*: node degree = 29) and DNA-directed RNA polymerase II subunit RPB1 (*RPII215*: node degree = 22) in clusters 1 and 2, respectively. Enrichment analysis indicated that each of these clusters had specific functional roles in their respective BP (Table S10 and S11). Most enriched GO processes in the largest cluster 1 were related to chemical synaptic transmission, ion transport, nervous system process, or development (Fig. 5 and Table S10). The smallest cluster 6 was enriched in processes related to organization, oogenesis, or regulation of growth, including others (Fig. 5 and Table S10). Meanwhile, numerous signaling pathways such as hippo signaling pathway – fly, AGE-RAGE signaling pathway, and phosphatidylinositol signaling system were found in cluster 1 (Fig. 5 and Table S11). Among the 257 nodes, 60 transcripts were commonly expressed in both genotypes, of which 25 transcripts that showed inconsistent expression profiles within the same genotype were excluded from the analysis. The remaining 35 transcripts were used for further analysis; four and six transcripts displayed consistent up and downregulation patterns in both genotypes, respectively (Fig. 5 and Table S12). On the other hand, the remaining 25 transcripts exhibited inconsistent expression patterns in both genotypes (Table S12). The enrichment analysis of GO terms of DETs up-regulated in KE1 and down-regulated in KB11 were related to membrane, cuticle, and muscle (Table S12). This appears to be associated with increased “Body Length” upon exposure to fish kairomones in KE1.

## Discussion

Predator-induced responses in *Daphnia* have been extensively investigated [36, 38]. However, there is still a lack of understanding regarding the genetic mechanisms that regulate the appropriate functional responses associated with phenotypic plasticity in response to predation risk. Additionally, the variation in life-history traits of different genotypes to predation stress in the same environment remains poorly understood from a genotype-specific perspective. Prey species exhibit various adaptations in response to predation, including alterations in their life history, behavior, and morphological and physiological traits [14]. For example, the life history showed the direction of early or late maturation and increased or decreased body size under vertebrate predation risk [9, 41]. Therefore, we selected two genotypes showing contrasting life-history traits to understand the variation in phenotypic plasticity of different genotypes in response to predation stress in the same environment from a genotype-specific perspective. The two genotypes of *D. galeata* examined in the study were similar in terms of life-history traits related to reproduction, but with respect to growth, one genotype increased body size, while the other decreased body size under predation risk, indicating contrasting adaptive strategies. To investigate the association between phenotypic variation and differentially expressed transcripts in response to predator-induced cues, the two *D. galeata* genotypes were subjected to gene expression profiling after exposure to fish kairomones, which mimics predation risk. In addition, selected DETs of both genotypes were functionally annotated, and GO, KEGG pathway, and interaction network of DETs analyses were performed to infer functions associated with phenotypic variation. Our results revealed that phenotypic plasticity is relevant to reproduction and growth under predation stress in *D. galeata*.

Similar to previous studies [12, 20], our results showed altered life-history traits related to reproduction and growth in both genotypes under fish predator stress. The genotype KB11 exhibited defense strategies to maintain the generation by advancing reproductive age, producing more offspring, and evading predators by reducing body size. Previous studies have reported the occurrence of early maturation and reduced *Daphnia* size in response to vertebrate predators [34, 35]. The ecological advantage of earlier reproduction before predation is the maintenance of the population numbers [34, 42]. If most individuals within a population produce relatively few offspring in a fish environment, it will result in fewer offspring in the next cohort, which could threaten the persistence of the entire population. Therefore, it is presumed to represent a strategy to maintain the generation by producing more offspring. Additionally, exposure to predatory fish kairomones can increase the reproductive rate in *Daphnia*, which can further enhance their fitness and ability to cope with predation pressure [34, 42]. In KE1, life-history traits related to reproduction changed similarly to KB11 in the presence of fish kairomones (Fig. 1A–D). However, growth-related life-history traits such as “Somatic Growth Rate” and “Body Length” increased, showing opposite patterns to KB11 (Fig. 1E and F). Exposure to predatory fish kairomones can increase body length in *Daphnia* as an adaptive response to predator-induced stress [33]. When *Daphnia* perceive the presence of predatory fish, they activate certain physiological, and behavioral mechanisms to increase their chances of survival. One of these mechanisms involves allocating more energy toward growth and development, which leads to an increase in body length. This response is thought to be mediated by the activation of certain signaling pathways and gene expression changes, which ultimately result in the up-regulation of growth-related processes. In addition, previous studies suggested that *Daphnia* under the threat of fish might grow faster during juvenile stages and then invest in high reproductive effort and fast clutch release once they reach maturity [33]. Furthermore, previous studies suggested that vulnerability to fish predation in *Daphnia* could be increased by a larger size at first reproduction, which can be interpreted as an ecologic cost (community trade-off) [43] of the defensive reaction, deviating from the classical prediction of decreased body size as a response to risk of fish predation [44, 45].

DE analysis was performed to understand the genetic mechanisms regulating the appropriate functional response associated with phenotypic plasticity in two *D. galeata* genotypes that exhibit contrasting phenotypic plasticity under predation stress. In the study, among the 194 DETs commonly identified in both genotypes, 85 DETs (13 up-regulated and 72 down-regulated) were consistent (Fig. 3) and likely commonly expressed in response to fish kairomones, regardless of differences in contrasting life-history traits in the two genotypes; with an increasing number of collected *D. galeata* genotypes, the number of consistent DETs will probably decrease. Among the 85 DETs, there was an up-regulated DET encoding *CT* (Table S7), an important regulator of the stress response and plays an important role in protecting cells from stress-induced damage in various species including *Drosophila* [46]. Moreover, one of the consistently up-regulated transcripts encoded *DPEP1* which is implicated in digestive functions (Table S7). Exposure to predator kairomones for one generation in *D. ambigua* led to an up-regulation of genes related to digestive functions [38]. In addition, it has also been demonstrated in *D. magna* that digestive enzymes such as peptidases affect juvenile growth rates [47], and that cyanobacterial protease inhibitors impair digestion by inhibiting gut proteases, causing significant damage to *D. magna* populations [48]. These studies corroborate our findings, suggesting that the activation of digestive functions improves digestion and feeding efficiency, which plays a vital role in resource allocation that comes with shifts in life history, such as producing a greater number of offspring. This is supported by the observed phenotypic variation, where both genotypes exhibited a higher number of offspring under predator stress. In addition, a transcript encoding vitellogenin (*VG*), a precursor to a major yolk protein, annotated with the Q868N5 in the UniProt database was up-regulated in both genotypes (Table S7). A previous study unveiled a set of DETs in *D. pulex*, where the up-regulated genes included vitellogenin genes, known for their significance in the response to invertebrate predation risk [36]. Increased vitellogenin expression seems to indicate early onset of vitellogenesis and increased fertility, and in the case of *D. galeata*, fish kairomone induction increases fertility, leading to the production of more offspring, which presumably requires a larger yolk pool. On the other hand, a transcript encoding vitellogenin-3 (*VIT-3*) annotated with the Q9N4J2 was down-regulated in both genotypes. This could be as it represents a trade-off between reproduction and survival. In the presence of a predator, it may be advantageous for *Daphnia* to prioritize survival over reproduction by allocating resources toward physiological processes that enhance their ability to escape or defend against predators, rather than investing in reproductive processes that require energy and resources. However, further studies are needed to understand the defense strategies mediated by the transcripts encoding the two vitellogenins, which exhibit opposite expression patterns.

The remaining 109 DETs showed inconsistent expression patterns depending on genotypes. Among them, *VINC* was up-regulated in KE1 but down-regulated in KB11. Previous studies have shown that *D. magna* exposed to cyclophosphamide, one of the most widely used drugs in cancer chemotherapy, showed upregulation of vinculin, a cytoskeletal protein involved in the regulation of focal adhesions and embryonic development [49, 50]. There is currently limited information on the specific function of *VINC* in the predator stress response of *Daphnia*, but it may play a role in increasing growth rate and fertility in response to predator cues, mediating changes in cell morphology, adhesion, and migration. However, further research is needed to determine the specific role of *VINC* in *Daphnia*’s predator stress response. The *OBST-E*, which belongs to the chitin binding peritrophin-A domain, was up-regulated in KE1 but down-regulated in KB11 (Table S7). Usually, growth-related chitin and cuticle genes were up-regulated by fish kairomone exposure in *Daphnia* [22, 32, 36]. In addition, this protein plays a role in the cuticle formation in *Drosophila* [51]. It has been reported that *Drosophila* evolved chitin-containing structures, such as cuticle or the peritrophic membranes, that serve to protect the body from hostile environments, and expression of *OBST-E* is involved in this [51]. *OBST-E* may increase the ability to survive by evolving the cuticle structure in Daphnia, which plays a protective role in the predator stress response. In summary, as shown by the phenotypic variation observations, genotype KE1 increased “Somatic Growth Rate” and “Body Length” under predator stress, while genotype KB11 decreased, which may be related to *VINC* and *OBST-E* protein. Moreover, 44 transcripts were down-regulated in KE1, but up-regulated in KB11 (Table S7). For example, *FLIL*, a conserved cytoplasmic protein found in a wide range of organisms, including fruit flies [52, 53], was down-regulated in KE1 but up-regulated in KB11. *FLIL* is known to play a role in cytoskeletal dynamics and actin remodeling, which are important processes in cellular morphology and motility [54]. However, given the limited research on *FLIL* expression in *Daphnia* and *Drosophila* under predation stress, it is difficult to explain the association of these DETs with the measured phenotypic variation. Further research is needed to fully understand the specific mechanisms regulating *FLIL* expression levels in response to predatory-induced signaling in *Daphnia*.

The two *D. galeata* genotypes found in the same environment exhibit a larger number of genotype-specific DETs compared to the common ones. Based on the six life-history traits measured, “Body Length” is the only trait showing the opposite phenotype in both genotypes (Fig. 1F). Growth-related muscle protein synthesis, which may affect body length, plays an important role in various metabolic processes, growth, and reproductive activities in crustaceans. The significance of muscle-specific genes and proteins in crustaceans has been highlighted by several research on invertebrates [55, 56]. In this study, up-regulated *MMP2* and *DOP2R*, related to growth, were only found in KE1. *MMP2* plays a role in extracellular matrix remodeling and is involved in several physiological processes including growth and development [57, 58]. *MMP2* expression is up-regulated in *D. pulex* under predator stress, indicating its potential involvement in predator-induced phenotypic plasticity [59]. Overall, *MMP2* appears to be an important mediator of predator-induced phenotypic plasticity in *Daphnia*, and may play a role in the regulation of growth and development under predator stress. *DOP2R* is a neurotransmitter known to be involved in various physiological processes, including growth and development [60]. Under predator stress, dopamine can have a role in modulating the response to stress and the allocation of resources toward to growth in *Daphnia*. A previous study has reported that dopamine application results in larger individuals at sexual maturity, where dopamine may affect cell proliferation and/or increase cell growth [60]. In contrast, two DETs belonging to the Lim domain, such as *PXN*, and *MLP84B*, were down-regulated in KB11, but not found in KE1 (Table S6). Lim domain proteins play crucial roles in various BP, including cytoskeleton organization, cell fate determination, and organ development [61]. Specifically, *MLP84B*, a cytoskeletal protein primarily expressed in *Drosophila* muscles, has been implicated in the regulation of actin filament organization and stability in muscle cells, which can affect muscle growth and function [62, 63]. This suggests that genotype KB11 represents an adaptive strategy to reduce body size to avoid being detected and eaten by predators by inhibiting growth by down-regulating Lim domain proteins involved in growth. However, further studies are needed to fully comprehend the specific mechanisms through which the described genotype-specific DETs are linked with the observed phenotypic variation in each genotype.

There are reports suggesting that signaling pathways regulate developmental changes during exposure to predators, which in turn affect reproduction and growth. The tachykinin (*TK*) receptor signaling pathway was only identified in the GO enrichment analysis of genotype KE1 (Table S8), which matures early, and has an increased body size. This pathway is involved in various physiological processes such as neurotransmission, muscle contraction, regulation of feeding behavior, and immune response in various organisms including *Drosophila* [64–66]. Especially, the *TK* in *Drosophila* brain is one of several factors that regulate the insulin-producing cells, which play important hormonal roles in the regulation of metabolic carbohydrates and lipids, as well as reproduction, growth, stress resistance, and aging [64–66]. Activation of the *TK* receptor in various environmental stress responses may lead to the release of neuropeptides, which modulate various physiological and behavioral responses such as increased heart rate, changes in locomotor activity, and altered feeding behavior. In addition, activation of the *TK* receptor signaling pathway has been shown to increase growth rates in *Drosophila* [64–66]. These responses are thought to be adaptive, allowing the flies to quickly respond to danger and increase their chances of survival. Overall, the *TK* receptor signaling pathway appears to be involved in promoting growth, and since the *TK* receptor signaling pathway was only activated in the genotype KE1, which showed increased growth rates under fish kairomone exposure, this is likely to have influenced growth. On the other hand, the cell differentiation, proliferation, and apoptosis-related pathways (hippo, FoxO, ECM-receptor interaction, and MAPK signaling pathways) and related to neuronal development such as Wnt signaling pathways were inhibited under fish kairomone exposure in only genotype KB11, which mature late and have decreased body size (Table S8). All these signaling pathways are involved in the regulation of embryonic development [67–69]. Hence, the disrupted regulation of these pathways could have detrimental impacts on embryonic development, potentially linking it to the impaired growth of genotype KB11 when exposed to fish kairomones. Among these pathways, a total of 28 DETs were mapped to the Wnt pathway, which was identified only in KB11 (Table S9). The Wnt pathway is a key signaling pathway that affects the late developmental stage of *Daphnia*; transcriptional profiling of *D. mitsukuri* exposed to fish kairomones revealed that it responds to predation risk by regulating the activity of the Wnt signaling pathway [70]. Thus, down-regulation of DETs within the Wnt pathway, which plays a pivotal role in animal development and growth [71, 72], may contribute to the smaller body size observed in genotype KB11. Further research is needed to fully elucidate the precise mechanisms and interactions within the Wnt pathway that contribute to body size determination in *Daphnia*.

While this study describes common and genotype-specific DETs in response to fish kairomone exposure, it is likely that DETs will change with additional populations and genotypes. Thus, to elucidate the association between the DETs of both genotypes and the observed phenotypic variations, it is crucial to generate RNA-seq data for additional *D. galeata* genotypes from the same and other populations. This approach would offer insights into shared or divergent life-history traits, allowing for a comprehensive understanding of the genetic basis underlying these phenotypic variations. Our findings could also be explained by the influence of epigenetic modifications, such as cytosine methylation [73]. In addition, in future studies, quantitative RT-PCR (qRT-PCR) should be performed on differentially expressed genes to find candidate genes and quantify the expression. Epigenetic factors can have a significant impact on phenotypic plasticity by modulating gene expression and influencing the response of an organism to environmental cues. In addition, intraspecific variation can have a significant impact on alternative splicing, and the diversity and abundance of isoforms can explain the intraspecific variation found under predation risk [74]. Alternative splicing is a key regulatory mechanism in eukaryotes that plays a pivotal role in transcriptional diversity and activity, significantly contributing to gene expression regulation. Long-read sequencing can serve to understand how different isoforms contribute to phenotypic variation in response to fish kairomone exposure. Our future research aims to elucidate the precise adaptive mechanisms underlying predator-induced responses.

## Conclusion

This study aimed to compare the predator-induced phenotypic variation and transcriptional profiles of in two contrasting *D. galeata* genotypes found in the Han River, Korea. The two *D. galeata* genotypes investigated in the study exhibited similarities in terms of life-history traits associated with reproduction; however, in terms of growth, one genotype increased body size, whereas the other genotype decreased it under fish kairomone exposure, indicating divergent adaptive strategies. To better understand the transcripts involved in phenotypic plasticity under predation stress, we conducted RNA-seq on their transcriptomes. Our results showed that the consistently up-regulated DETs in both genotypes encoded proteins involved in digestion and reproduction, which can be interpreted as life-history traits related to reproduction. Furthermore, among the DETs exhibiting inconsistent expression patterns in both genotypes, genes associated with growth were up-regulated in KE1 but down-regulated in KB11, suggesting a potential link with life-history traits associated with growth. The present findings are in line with the results obtained from GO, KEGG, and network analyses, which demonstrated the involvement of various processes associated with reproduction and growth. Nevertheless, additional research is required to gain a comprehensive understanding of the precise mechanisms through which the described DETs result in the observed phenotypic variation in each genotype. Our analysis allows for a better understanding of the adaptation mechanisms related to reproduction and growth of two Korean *D. galeata* genotypes under predation stress.

## Methods

### Sample collection, culture, and molecular identification

Two *D. galeata* genotype samples were collected in the same environment (37°30’46.0” N, 126°59’53.7” E) from the Han River, Korea, on June 2020, using a 55 μm mesh sized Kitahara Zooplankton net (Samjee-tech, Korea). After transferring the collected samples to the laboratory, each egg-bearing female individual was transferred to a separate beaker for parthenogenetic reproduction. *D. galeata* individuals were cultured under laboratory conditions (20℃, 16 h light/8 h dark cycle, ISO medium); 1.0 mg C L^− 1^*Chlorella vulgaris* was used as food source once daily. The two genotypes examined were identified by mitochondrial cytochrome oxidase I (*cox1*) and NADH dehydrogenase subunit 2 (*nd2*) gene sequence analyses [28, 30]. As a result, we identified only two different genotypes with sequence differences in the *cox1* and *nd2* genes. In addition, the haplotype network based on *nd2* gene revealed genotype KE1 belonged to clade D and KB11 to clade B1 (Figure S3).

### Experimental design and life-history experiment

This study was conducted using two *D. galeata* genotypes (KE1 and KB11) cultured for three years prior to this experiment and had established clonal lines. To mimic fish predation, *D. galeata* was exposed to a kairomone-enriched medium produced by growing approximately 20 freshwater mandarin fish (*Siniperca scherzeri*) in 100 L of water. This medium was obtained from the aquaria where freshwater mandarin fish were raised. The medium in the fish aquaria was changed three times a week, and the medium containing kairomones was prepared by filtering it through a 0.45-µm-pore-sized, cellulose acetate membrane (Hyundai-Micro, Korea) and mixing it the ISO medium at a 1:2 ratio. The ratio of fish kairomone medium was set as the ratio at which the changes occur in life-history traits while ensuring the survival rate of *D. galeata* after several trials. The experimental design and procedures based on the previous research [32]. To minimize inter-individual variances, both genotypes were bred for two consecutive generations in a kairomone-free medium (control group) and fish kairomone medium (experimental group) prior to the experiment. After culturing until the second generation, 20 egg-bearing females of each genotype from the medium were considered as grandmothers (F0) to the experimental animals (F2). According to Tams et al., *D. galeata* showed strong changes in life-history traits after three generations of fish kairomone exposure [9]. To investigate the long-term effects of fish kairomones on transcript expression levels, the experiment lasted 14 days for each experimental individual. During this time, life-history traits related to reproduction such as “Age at First Reproduction,” “Number of Offspring First Brood,” “Total Number of Broods,” and “Total Number of Offspring” were recorded. In addition, life history variables related to growth such as “Somatic Growth Rate” and “Body Length” were estimated. The “Age at First Reproduction” refers to the day when the first neonates were released from the brood pouch. The “Total Number of Offspring” was calculated by the number of summing offspring produced by each individual during the induction experiment. Additionally, the “Somatic Growth Rate” was measured by subtracting the body length of the neonate at the start of the experiment (t0) from that of the adult at the end of the experiment (t14) and dividing it by the total duration of the experiment (14 days). The measurement of body length excluded the spine itself and was taken from the top of the head, passing through the middle of the eye, ending at the ventral basis of the spine [9]. The individuals were observed using CX22LED microscope (Olympus, Japan), and photographed using eXcope T500 microscope camera (DIXI Science, Korea). The statistical analyses for life-history traits were conducted using the aov() function in R version 4.2.2 [75, 76]. The egg-bearing females (F2) were pooled (n = 20) after 14 days; three biological replicates per experimental condition (control and fish kairomone exposed) were used for the two genotypes, resulting in a total of 240 individuals (two genotypes × two conditions × 20 individuals × three biological replicates; Figure S4). The samples were preserved in Qiagen RNAprotect tissue reagent (Qiagen, Valencia, CA, USA) at 4ºC overnight and subsequently stored at -80ºC until RNA extraction.

### Data collection and analysis

#### RNA preparation and high-throughput sequencing

Since we could not obtain a sufficient amount of RNA from one individual, we pooled the experimental individuals. As embryos contain high amounts of RNA and can perceive predator cues, we accounted for the stage of egg development within the brood pouch [77]. Because sampling females in the inter-molt stage can ensure stable gene expression, only egg-bearing experimental females were pooled [77].

Total RNA was isolated from pools of 20 egg-bearing adults using the Qiagen RNeasy Plus Universal Mini Kit (Qiagen, Valencia, CA, USA) following manufacturer’s instructions after homogenizing with a disposable pestle and homogenizer. Samples were stored at − 80ºC until RNA isolation. RNA quality was assessed using an Agilent 2100 bioanalyzer (Agilent Technologies, Amstelveen, The Netherlands), and RNA quantification was conducted using an ND-2000 Spectrophotometer (Thermo Inc., DE, USA) for 12 samples (two genotypes × two conditions × three biological replicates). Libraries were constructed from the total RNA using the NEBNext Ultra II Directional RNA-seq Kit (New England BioLabs, Inc., UK). The Poly(A) RNA Selection Kit was employed to isolate mRNA (Lexogen, Inc., Austria). The isolated mRNAs were utilized for cDNA synthesis and shearing following manufacturer’s instructions. Indexing was conducted utilizing Illumina indexes 1–12, followed by enrichment by PCR. Subsequently, the libraries were assessed using the TapeStation HS D1000 Screen Tape (Agilent Technologies, Amstelveen, The Netherlands) to determine the mean fragment size. Library quantification was conducted using a library quantification kit on a StepOne Real-Time PCR System (Life Technologies, Inc., USA). High-throughput sequencing was conducted using the NovaSeq 6000 (Illumina, Inc., USA) platform, generating paired-end reads of 101 base pairs from each end.

**RNA-seq quality control and*de novo*transcriptome assembly**.

A quality control of raw sequencing data was performed using FastQC v.0.11.9 [78]. Adapter and low-quality reads (< Q20) were removed using Trimmomatic v.0.36 with the following parameters: ILLUMINACLIP: TruSeq3-PE.fa:2:30:10 TRAILING: 20 SLIDINGWINDOW: 4:15 MINLEN: 36 [79]. After trimming, the read quality was checked again with FastQC for successful adapter removal. Since the annotation of the reference genome of *D. galeata* is incomplete and insufficient for further analysis, a *de novo* approach was employed to assemble the RNA-seq reads. In addition, a *de novo* assembly was performed to use the transcripts found in both genotypes after fish kairomone exposure. The *de novo* transcriptome assembly was performed by combining the resulting quality-filtered reads of RNA-seq data of two genotypes using Trinity v.2.14.0 default parameters [80].

### Assembly assessment and unigene calculation

To calculate the assembly statistics, the TrinityStats.pl script from the Trinity pipeline was utilized. The transcriptome completeness of all datasets was assessed using BUSCO v5.3.2 against the Arthropoda dataset (Arthropoda Odb10), composed of 1013 single-copy ortholog genes with default parameters [81]. To eliminate sequence redundancy, unigenes with 95% similarity were calculated using CD-Hit [82]. The reads were mapped back to the unigenes using RNA-seq by Expectation Maximization [83].

### Functional annotation of unigenes

The TransDecoder pipeline with default parameters was employed to predict potential coding regions and ORFs [84]. All ORFs analyzed in this study were required to be ≥ 100 amino acids in length. BLASTp analysis was conducted on the unigenes using an e-value cutoff of 1e-5 against the UniProt database [85, 86]. The top hit for each query sequence was selected for transcriptome annotation and further characterization. HMMER v.3.1b2 was utilized against the Pfam database to identify protein domains [87, 88]. The online functional annotation tool, eggNOG-mapper, was used to determine orthologous groups [89]. GO terms enrichment of the DETs was analyzed using the ShinyGO v.0.61 [90]. Enrichment of KEGG pathways of the DETs was analyzed using KEGG mapper and KOBAS v.3.0 web-based tools [91, 92]. Only enriched categories showing a corrected *p*-value < 0.05 were selected.

### Differential transcript expression analysis

DE analysis was performed at the isoform level using the DESeq2 package for R v4.1.3 within the Trinity pipeline [75, 93]. Results were subjected to post hoc filtering to reduce the false discovery rate by applying an adjusted *p*-value (padj) < 0.05 [94]. Additionally, a fold change ≥ 2 (log2FC ≤ -1 or log2FC ≥ 1) was used to filter the results. To confirm the quality of the triplicated samples, PCA was performed with DEseq2 and ggplot2 [95]. The number of shared transcripts between genotypes was visualized using the web-based tool Venn (https://bioinformatics.psb.ugent.be/webtools/Venn/).

### Network analysis and community detection

To predict the interactions between these DETs, the annotated UniProt IDs of the DETs were mapped using Cytoscape stringApp v.1.5.1 using *Drosophila melanogaster* (fruit fly) as reference organism [96]. The interaction network of the DETs was visualized and further investigated using Cytoscape v.3.7.0 [97]. Clusters with < 10 nodes were excluded from the analysis. Node degree distribution and community structure were assessed using the NetworkAnalyzer and GLay plugins of Cytoscape, respectively [98]. The modular structure of the interaction network was identified by using the GLay implementation of the fast-greedy algorithm within the clusterMaker plugin of Cytoscape [99]. This algorithm identifies clusters by iteratively removing edges from the network and then reevaluating the connected nodes [100].

### Electronic Supplementary Material

Below is the link to the electronic supplementary material.

Supplementary Material 1

Supplementary Material 2

Supplementary Material 3

Supplementary Material 4

Supplementary Material 5

Supplementary Material 6

Supplementary Material 7

Supplementary Material 8

Supplementary Material 9

## Data Availability

Sequence reads generated and/or analyzed from this study are available on the Sequence Read Archive (SRA) SRR24475174 ~ SRR24475185 under BioProject PRJNA970532 (https://www.ncbi.nlm.nih.gov/bioproject/PRJNA970532).

## Abbreviations

DET: Differentially Expressed Transcript
DE: Differential Expression
GO: Gene Ontology
KEGG: Kyoto Gene and Genome Encyclopedia
ORF: Open Reading Frames
PCA: Principal Component Analysis
BP: Biological Process
MF: Molecular Function
CC: Cellular Component
coxI: cytochrome oxidase I
nd2: NADH dehydrogenase subunit 2
