# Supplementary material for: Comparative transcriptome analysis of two Daphnia galeata genotypes displaying contrasting phenotypic variation induced by fish kairomones in the same environment of the Han River, Korea
Source: BMC Genomics. 2023 Oct 2;24:580. doi: 10.1186/s12864-023-09701-x (PMC10544471; doi:10.1186/s12864-023-09701-x)
Supplement: Supplementary file 1 — Supplementary Material 1 [file 12864_2023_9701_MOESM1_ESM.docx]

**Supplementary Information**

**Comparative transcriptome analysis of two *Daphnia galeata* genotypes displaying contrasting phenotypic variation induced by fish kairomones in the same environment of the Han River, Korea**

Tae-June Choi^1^, Seung-Min Han^1^, Adeel Malik^2^, Chang-Bae Kim^1,^ *

^1^ Department of Biotechnology, Sangmyung University, Seoul 03016, Republic of Korea

^2^ Institute of Intelligence Informatics Technology, Sangmyung University, Seoul 03016, Republic of Korea

Corresponding author: Chang-Bae Kim (evodevo@smu.ac.kr)

**Table S1**. Statistics for the *D. galeata* transcriptome dataset.

| Genotype | | Total number of raw reads pairs | Total number of clean reads pairs | Read length (nt) | GC contents (%) |
| --- | --- | --- | --- | --- | --- |
| KE1-  control | 1 | 57,713,081 | 56,438,930 | 36-101 | 46 |
|  | 2 | 57,982,530 | 56,593,269 | 36-101 | 47 |
|  | 3 | 55,095,145 | 53,688,100 | 36-101 | 48 |
| KE1-  fish | 1 | 55,663,786 | 54,516,209 | 36-101 | 46 |
|  | 2 | 57,955,257 | 56,799,236 | 36-101 | 46 |
|  | 3 | 57,521,753 | 56,487,670 | 36-101 | 46 |
| KB11-  control | 1 | 68,537,278 | 67,143,026 | 36-101 | 48 |
|  | 2 | 57,656,766 | 56,484,946 | 36-101 | 48 |
|  | 3 | 68,916,607 | 67,436,216 | 36-101 | 46 |
| KB11-  fish | 1 | 57,731,594 | 56,679,079 | 36-101 | 48 |
|  | 2 | 57,421,146 | 56,130,691 | 36-101 | 48 |
|  | 3 | 52,619,624 | 51,338,497 | 36-101 | 47 |

**Table S2.** Transcriptome datasets statistics and assembly.

| Description | Statistics |
| --- | --- |
| Number of transcripts | 186,084 |
| Percent GC content (%) | 41.06 |
| Contig N50 | 4,588 |
| Median contig length (bp) | 700 |
| Average contig length (bp) | 1,877.92 |
| Total assembled bases (bp) | 349,450,193 |
| Total number of unigenes | 135,611 |
| Percent unigenes GC content (%) | 40.7 |
| Longest unigene (bp) | 28,597 |
| Shortest unigene (bp) | 181 |

N50. Sequence length of the shortest contig at 50% of the total assembly length.

**Table S3**. Results of BUSCO analysis.

| Description | Total Number | Rate (%) |
| --- | --- | --- |
| Total BUSCO groups searched | 1,013 | 100.0 |
| Complete BUSCOs | 1,003 | 99.1 |
| Complete and single-copy BUSCOs | 111 | 11.0 |
| Complete and duplicated BUSCOs | 892 | 88.1 |
| Fragmented BUSCOs | 5 | 0.5 |
| Missing BUSCOs | 5 | 0.5 |

**Table S5.** Summary of unigene annotation results.

| Description | Number of unigenes | Percentage (%) |
| --- | --- | --- |
| Total number of longest ORF unigenes | 89,738 | |
| Annotated in UniProt | 56,306 | 62.74 |
| Annotated in Pfam | 67,873 | 75.63 |
| Annotated in eggNOG | 59,682 | 66.50 |
| Annotated in KEGG | 1,405 | 1.56 |
| Total of annotated unigene (unique) | 74,082 | |


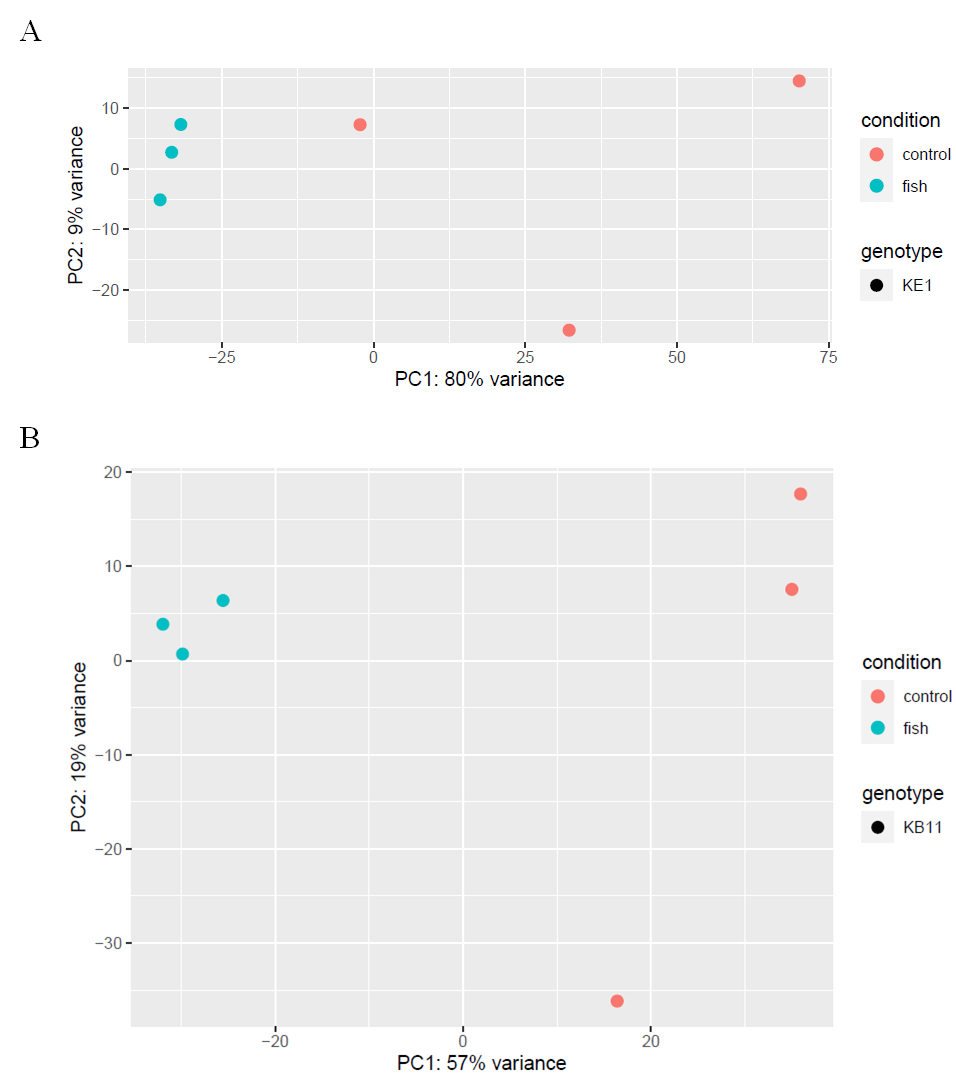


**Figure S1.** Principal component plot of biological RNA-seq samples in *D. galeata*. A, genotype KE1. B, genotype KB11. Red and cyan circle indicates the control (without fish kairomones) and experimental (fish kairomone-exposed) group, respectively.


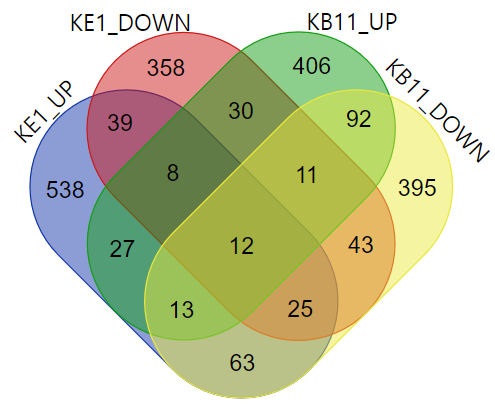


**Figure S2.** Venn diagram showing the unique number of differentially expressed transcripts identified from each genotype in *D. galeata*. KE1, genotype KE1. KB11, genotype KB11. UP, up-regulated transcripts. DOWN, down-regulated transcripts.


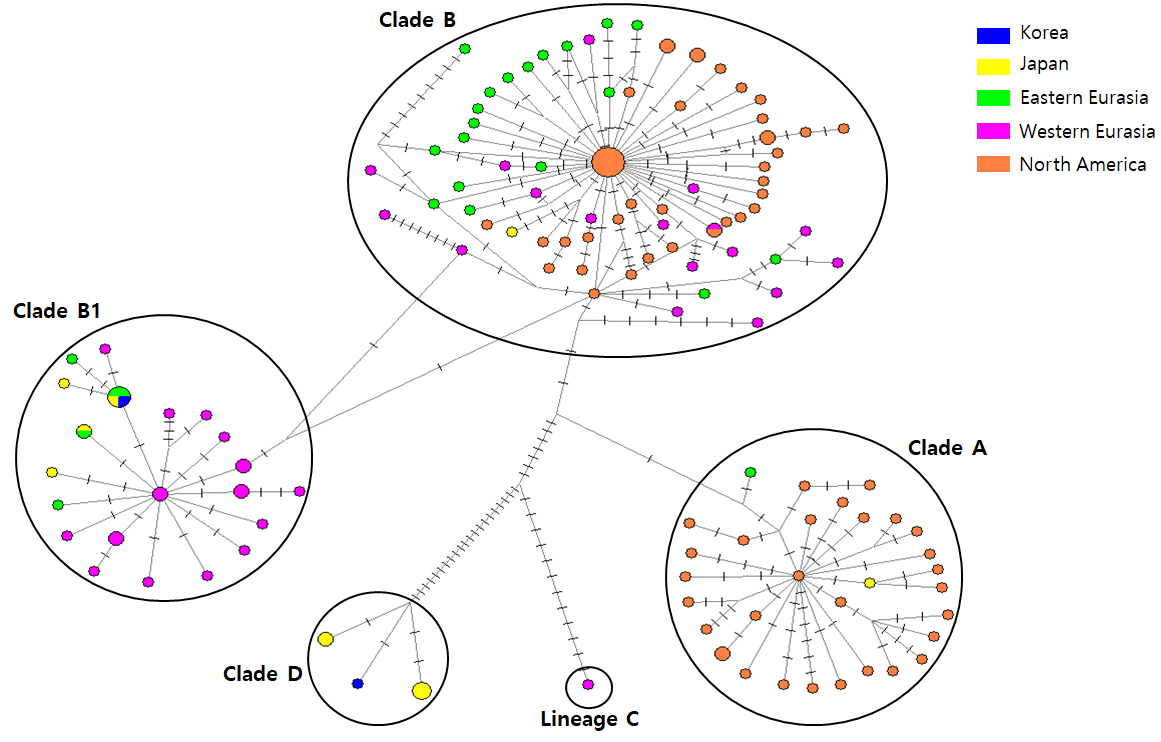


**Figure S3.** Median-Joining network of haplotypes based on the variation of the mitochondrial *nd2* gene observed in the Holarctic populations of *Daphnia galeata*. The blue color indicates two genotypes collected from Han River, Korea analyzed in this study. Mutated positions are shown as branch lines.


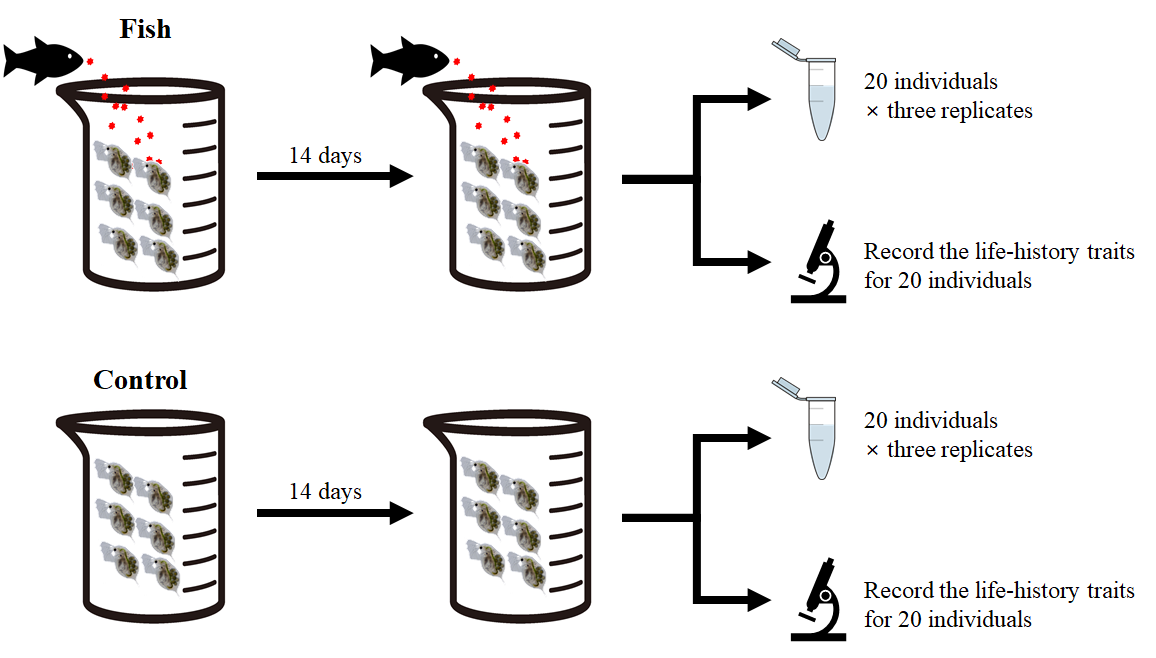


**Figure S4.** The schematic diagram depicts the fish kairomone induction experiment in *D. galeata*. RNA-seq data were generated from three biological replicates, each consisting of 20 individuals from both control and fish kairomone treatments in both genotypes. These 20 individuals were used to record the life-history traits.
